# Insights into the Molecular Mechanisms Mediating Extravasation in Brain Metastasis of Breast Cancer, Melanoma, and Lung Cancer

**DOI:** 10.3390/cancers15082258

**Published:** 2023-04-12

**Authors:** Rama Alsabbagh, Munazza Ahmed, Mohammad A. Y. Alqudah, Rifat Hamoudi, Rania Harati

**Affiliations:** 1 Department of Pharmacy Practice and Pharmacotherapeutics, College of Pharmacy, University of Sharjah, Sharjah 27272, United Arab Emirates; u18200545@sharjah.ac.ae (R.A.); u21104594@sharjah.ac.ae (M.A.);; 2 Research Institute for Medical and Health Sciences, University of Sharjah, Sharjah 27272, United Arab Emirates; 3 Department of Clinical Pharmacy, Faculty of Pharmacy, Jordan University of Science and Technology, Irbid 22110, Jordan; 4 Clinical Sciences Department, College of Medicine, University of Sharjah, Sharjah 27272, United Arab Emirates; 5 Division of Surgery and Interventional Science, University College London, London W1W 7EJ, UK

**Keywords:** brain metastasis, molecular mechanisms, lung cancer, breast cancer, melanoma

## Abstract

**Simple Summary:**

Despite the advancements in systemic treatments, the incidence of brain metastasis (BM) is increasing. BM is a multi-step cascade. One critical event in BM is the extravasation of cancer cells through the blood–brain barrier (BBB), a physiological barrier located on the brain microvasculature, persevering the cerebral homeostasis. The molecular mechanisms driving BM, particularly those driving extravasation, remain poorly understood. A better understanding of these mechanisms will allow the identification of potential molecular targets that can be used to treat or prevent BM. In this review, we summarize the molecular events that occur during extravasation in three types of cancer most likely to develop brain metastasis: breast cancer, melanoma, and lung cancer. Specifically, we summarize and compare the current knowledge on the molecular mechanisms mediating steps of extravasation, as well as the mechanisms inducing alterations in the barrier.

**Abstract:**

Brain metastasis is an incurable end-stage of systemic cancer associated with poor prognosis, and its incidence is increasing. Brain metastasis occurs through a multi-step cascade where cancer cells spread from the primary tumor site to the brain. The extravasation of tumor cells through the blood–brain barrier (BBB) is a critical step in brain metastasis. During extravasation, circulating cancer cells roll along the brain endothelium (BE), adhere to it, then induce alterations in the endothelial barrier to transmigrate through the BBB and enter the brain. Rolling and adhesion are generally mediated by selectins and adhesion molecules induced by inflammatory mediators, while alterations in the endothelial barrier are mediated by proteolytic enzymes, including matrix metalloproteinase, and the transmigration step mediated by factors, including chemokines. However, the molecular mechanisms mediating extravasation are not yet fully understood. A better understanding of these mechanisms is essential as it may serve as the basis for the development of therapeutic strategies for the prevention or treatment of brain metastases. In this review, we summarize the molecular events that occur during the extravasation of cancer cells through the blood–brain barrier in three types of cancer most likely to develop brain metastasis: breast cancer, melanoma, and lung cancer. Common molecular mechanisms driving extravasation in these different tumors are discussed.

## 1. Introduction

Brain metastasis (BM) is an incurable end-stage of cancer. It is associated with poor prognosis and its incidence is increasing due to the advancements of systemic treatments and the limited ability of drugs to cross the blood–brain barrier (BBB) and reach the brain [1]. BM preferentially originates from lung cancer (50–60%), breast cancer (20–30%), and melanoma (5–10%) [2,3].

BM occurs through a multistep cascade by which cancer cells disseminate from the primary tumor site to the brain. This metastatic cascade involves epithelial–mesenchymal transition (EMT), primary tissue invasion, intravasation in the systemic bloodstream, and survival in blood circulation, followed by extravasation through the BBB and homing into the brain where the development of micro-metastatic lesions takes place and progresses to form macroscopic tumors [4]. Once in the brain, malignant cells adapt to the brain microenvironment and exploit their advantages to proliferate [4]. The BBB further protects the resulting metastatic tumors against therapeutic treatments and from immune surveillance [5,6,7,8].

A key step in the brain metastatic cascade is the extravasation of tumor cells through the BBB. However, this step remains to be fully characterized. A better understanding of the molecular mechanisms contributing to extravasation may open avenues to design new therapeutic and preventive strategies. In this review, we summarize the potential molecular events that occur during the extravasation of three types of cancer most likely to develop BM: breast cancer, melanoma, and lung cancer.

## 2. Extravasation of Tumor Cells through the Blood–Brain Barrier as A Key Step in Brain Metastasis

Extravasation of tumor cells through the BBB is a key step of the metastatic cascade in which circulating cancer cells pass through the BBB to reach the brain [9].The BBB is a multi-cellular structure located in the brain microvessels at the interface between the peripheral blood and the brain [7,10]. It controls the flow of cells, molecules, and ions between the blood and the central nervous system (CNS). Specifically, the barrier regulates the entry of nutrients and necessary elements, and limits the passage of xenobiotics as well as potentially neurotoxic substances from the blood into the brain. By this function, the BBB helps in maintaining the homeostatic micro-environment of the CNS needed for the proper functioning of the neuron [11,12]. Structurally, the BBB consists of the brain endothelial cells (BECs) characterized by the lack of fenestration and their strong attachment to each other by inter-endothelial junctions, pericytes, a basement membrane, and an astrocytic end-feet ensheathing of the brain microvessels [13]. The brain endothelium (BE) forms the wall of the brain microvessels where the cells are held together through junctional protein complexes composed of tight junctions, adherens junctions [13,14], and gap junctions that facilitate cell to cell communication [15]. The tight junctions consist of transmembrane proteins, including occludin, claudins, and junction adhesion molecules (JAM), as well as cytoplasmic accessory proteins; for example, cingulin and zonula occluden proteins (ZO-1,2,3) responsible for linking the transmembrane proteins to the actin cytoskeleton protein to sustain the endothelium integrity [16]. The presence of tight junctions in the brain strictly prevents the paracellular diffusion of cells and molecules into the brain [14], while the adherens junctions keep adjacent cells and the junctional complexes together [17]. The adherens junctions consist of transmembrane proteins, including the vascular endothelial cadherin (VE-cadherin) and the platelet endothelial cell adhesion molecule 1 (PEC; as for the differences, few studies have investigated the unique molecular mechanisms mediating extravasation in a specific type of AM-1). These transmembrane proteins are coupled with the actin cytoskeleton through the cytoplasmic catenin (α, β, γ -catenin) proteins [16,18,19].

During extravasation, cancer cells with high metastatic capabilities succeed in surpassing the BBB to reach the brain. Extravasation occurs through several steps: cancer cell rolling through the BE, firm adhesion, and trans-endothelial migration (TEM) through the BBB [20,21]. For the extravasation to take place, cancer cells slow down while moving through the blood flow, arrest within the capillaries according to their sizes, and then roll alongside the surface of the BE via multiple receptor–ligand interactions with the BEC [4,22]. Tumor cells then induce alterations in the BBB and transmigrate through the barrier to reach the brain [17].

### 2.1. Molecular Mechanism Mediating Extravasation in Breast Cancer Brain Metastasis

Breast cancer is the most common type of cancer among women worldwide [23]. Early diagnosis of breast cancer through mammography screening allows more chances for successful treatment and subsequently reduces death rates [24]. Breast cancer can be classified according to the receptor expression in the breast cancer cells: hormone receptor-positive, which involves expression of estrogen (ER) or progesterone receptors (PR); human epidermal growth factor receptor 2 (HER2)-positive, which represents over-expression of human epidermal growth factor receptor-2; and the triple negative breast cancer that is negative for ER, PR, and HER2 receptors [4,25]. Around 20–30% of women diagnosed with breast cancer develop brain metastases [2]. Patients diagnosed with HER2-positive or triple negative breast cancer have a higher risk of BM compared to hormone-positive patients [26]. Once diagnosed with BM, the median survival range of breast cancer patients falls between 3.4 and 25.3 months, with triple negative breast cancer having the poorest survival rate and HER2-positive patients the longest survival rate [1].

#### 2.1.1. Factors Mediating Rolling and Firm Adhesion of Breast Cancer Cells to the Brain Endothelium

Molecular interactions between breast cancer cells and BECs are essential in initiating the extravasation and maintaining the metastasis process. Early in the extravasation step, circulating cancer cells roll along the BE, navigate for suitable sites to cross the BBB, and then form transient interactions with the BEC [27]. Rolling is promoted by adhesion glycoprotein molecules, such as selectin, expressed on the BEC. Particularly, the E-selectin interacts with its ligands expressed on breast cancer cells, including platelet selectin glycoprotein ligand-1 (PSGL-1), cluster of differentiation 24 (CD24), sialyl-Lewis X (sLex), and mucin-1 (MUC1) to allow rolling [28,29]. Once the initial interaction of adhesion is established and the rolling events are decelerated, a firm and stable adhesion of the breast cancer cells to the BE is subsequently established. The firm adhesion is promoted by the endothelial E-selectin binding to its ligand (CD44) expressed in estrogen-negative (ER-) breast cancer cells [30]. Hyaluronan (HA), a primary ligand of CD44 [31], was shown to promote tumor cell adhesion, disruption, and migration through the BE in vitro as well as the extent of BM in vivo [32]. CD44 knockdown in brain seeking triple negative breast cancer cell line (MDA-MB-231-BR) reduced the pericellular HA coat on cancer cells, and consequently, their adhesion and invasion through the BE, suggesting the interaction between HA and CD44 as a potential therapeutic target of BM [32]. In addition, the under-glycosylated form of MUC1 expressed on breast cancer cells binds to E-selectin, whereby it interacts with intercellular adhesion molecule-1 (ICAM-1), a member of immunoglobulin superfamily (IgSF) expressed on endothelial cells, to facilitate rolling and firm adhesion of breast cancer cells to the BE [33,34]. Furthermore, ICAM-1 and vascular cell adhesion molecule-1 (VCAM-1) expressed on the BE bind to lymphocyte function-associated antigen 1 (LFA-1, also known as integrin αLβ2) and very late antigen-4 (VLA-4, also known as integrin α4β1) expressed on tumor cells, respectively. Activated leukocyte cell adhesion molecule (ALCAM) is another member of IgSF that is found to be upregulated in both endothelial cells and breast cancer cells, leading to homophilic ALCAM/ALCAM interactions. Blocking VLA-4-1 and ALCAM expressed on tumor cells was shown to significantly reduce BM through decreasing tumor cell adhesion and subsequent extravasation across the BE [28]. Moreover, integrin β4 mediate indirect adhesion to the BE by driving HER2+ breast cancer cells to induce vascular endothelial growth factor (VEGF) expression, which in turn degrades tight and adherens protein junctions of BE (ZO-1, VE-cadherin) [35]. The molecular mechanism by which VEGF induces alteration in the brain endothelium during extravasation remains to be fully characterized. However, there could be a possible role of metalloproteinase as previous studies have shown that VEGF causes the disruption of the BBB through metalloproteinase-9 (MMP-9) in a rat model of traumatic brain injury [36].

Interestingly, pericytes, which are important components of the BBB, have been shown to play a crucial role in the development of brain metastasis [37]. Few studies have investigated the role of pericytes in extravasation. It was found that brain pericytes exert a chemoattractant effect on breast cancer cells and establish direct contacts with them. Pericytes were shown to secrete high amounts of extracellular matrix proteins and enhance the adhesion of both melanoma and triple negative breast cancer. In addition, pericytes-secreted insulin-like growth factor 2 (IGF2) was found to have a very significant pro-proliferative effect on mammary carcinoma. Blocking IGF2 signaling by picropodophyllin reduced the proliferative effect of pericytes on breast cancer cells and reduced the size of brain tumors in mice inoculated with triple negative breast cancer [38]. Furthermore, disseminated cancer cells employ cell adhesion molecule L1 (L1CAM) to spread on capillaries and activate the mechanotransduction effectors Yes-associated protein (YAP) and myocardin-related transcription factor (MRTF). In another study, disseminated breast cancer cells that employ L1CAM (cell adhesion molecule L1) to spread on capillaries were shown to displace resident pericytes, which also use L1CAM for perivascular spreading. L1CAM activates YAP by engaging β1 integrin and integrin-linked kinase (ILK), allowing the outgrowth of metastasis-initiating cells in the metastatic site [39]. These findings shed light on pericytes in BM as potential therapeutic targets in brain metastatic diseases. However, more studies are still needed to better understand the role of pericytes in brain metastasis, particularly in the extravasation step.

#### 2.1.2. Factors Affecting the BBB Permeability in Breast Cancer Brain Metastasis

Once firm adhesion is established between metastatic breast cancer cells and the BE, disruptions or remodeling in the inter-endothelial junctions enhance the BE permeability, resulting in an altered vasculature known as the blood–tumor barrier (BTB) [40], and allow metastatic cells to transmigrate between the BEC and cross the BBB [41]. Breast cancer cells preferentially cross the BBB through a paracellular pathway rather than the transcellular route. In the paracellular route, cancer cells first interact with tight junctional proteins of endothelium, then with adherens junctional proteins, facilitating the opening of the intercellular junctions and consequently permitting the passage of cancer cells between adjacent endothelial cells. The junctional proteins of BE are partially degraded due to the firm adhesion of metastatic cancer cells, while they are completely disrupted during the last step in the extravasation event TEM [4]. Aside from the paracellular migration that involves the remodeling of junctional endothelial proteins, breast cancer cells have been shown to transmigrate into BECs while maintaining the ECs completely intact, indicating the possible utilization of a transcellular route through individual BECs [42].

Several metalloproteinases released during extravasation of breast cancer cells have been found to play a key role in disrupting junctional proteins of BE. Matrix metalloproteinases (MMPs) are multi-functional enzymes in inflammation. The role of MMPs in tumor progression and metastasis has been well documented. In extravasation, MMPs contribute to the disruption of junctional proteins. MMP1 overexpression has been linked to the BM of breast cancer, with MMP1 being upregulated in brain metastatic breast cancer cells compared to their parental cell lines. MMP1 facilitates cancer cell transit across the endothelium of the brain and increases BBB permeability by degrading the inter-endothelial junctions (claudin-5 and occludin) [43]. Consequently, knocking down MMP1 expression significantly decreases BM [44]. MMP9 and ADAM8 (a disintegrin and metalloprotease) co-regulation has been demonstrated to promote breast cancer BM, with ADAM8 reported to upregulate MMP9 and the shedding of P-selectin glycoprotein ligand (PSGL-1) from breast cancer cells. Shedding PSGL-1 increases invasiveness and promotes TEM [45]. Indeed, ADAM proteases interact with integrins to mediate a barrier function by active proteolysis. In addition, integrin-mediated signals enhance extracellular activities of MMPs which promotes a loss of the integrity of the basement membrane and allow the invasion of tumor cells via proteolytic breakdown of tight junctions and extracellular matrix proteins [45].

In an in vitro BBB model, breast cancer cells were found to secrete the proinflammatory neuropeptide Substance P (SP) that induces the secretion of tumor necrosis factor alpha (TNF-a) and angiopoietin-2 (Ang-2), leading to the redistribution of tight junction proteins ZO-1 and claudin-5 and resulting in an enhanced permeability of BE [46]. Disruption of the tight junction proteins (ZO-1 and claudin-5) by Ang-2 was shown to be partially dependent on the level of VEGF expressed by the breast cancer cells. Combination treatment of an Ang-2 inhibitor with a VEGF inhibitor was suggested as a possible therapeutic treatment [47]. Another study showed that VEGF highly expressed in breast cancer cells promotes TEM across the BBB by causing rearrangement of the cytoskeleton, which increases the BBB permeability [48].

Furthermore, during the adhesion and transmigration of breast cancer cells, there is degradation of endothelial surface glycocalyx (ESG) and disruption of ZO-1. In addition, cysteine proteases such as the cathepsin S expressed by breast cancer cells mediate proteolytic cleavage of JAM-B to induce the TEM of breast cancer cells [21]. Inhibition of cathepsin S was shown to reduce TEM in vitro [49].

Heparanase (HPSE) is an endoglycosidase enzyme that also increases the BBB permeability by disrupting endothelial junctions during breast cancer BM. HPSE acts as a pro-angiogenic and pro-metastatic agent by cleaving the heparan sulfate side chains of HSPG found in the extracellular matrix (ECM), causing the ECM to disintegrate and remodel [50,51]. This HPSE expression and activity was inhibited by the upregulation of micro-RNA-1258 (miR-1258) in brain metastatic breast cancer cells [52]. HPSE also mediates breast cancer BM by regulating proteoglycans in an enzymatically independent manner. It can induce guanine nucleotide exchange factor-H1 (GEF-H1) signaling that contributes to cytoskeletal dynamics, cellular adhesion, and BBB transmigration. Exogenous HPSE treatment promotes adhesion through inducing the expression of integrin β1 by brain metastatic triple negative breast cancer cells and the expression of VCAM-1 in BECs [53].

Disruption of BBB during breast cancer BM can be additionally promoted by the loss of a major facilitator superfamily domain 2a (Mfsd2a) expression in tumor endothelium. Mfsd2ais a transporter found in the brain needed for the uptake of fatty acids, in particular the docosahexaenoic acid (DHA) [54]. DHA (omega 3) is an essential lipid in the brain that is obtained from dietary sources and is needed for maintaining normal brain function. Multiple studies have revealed its anti-cancer effect during cancer progression and metastasis [55,56]. Downregulation of Mfsd2a impairs the transport of essential fatty acids (DHA) and affects the level of lipid metabolites, consequently promoting BM by increasing BBB permeability [54].

It is important to note that the BBB permeability is differently affected based on the molecular type of breast cancer cells. For instance, a small-sized retrospective cohort study showed that the disruption of BBB is linked with the BM of triple negative breast cancer but not with HER2-positive breast cancer. To assess the integrity of BBB in resected BM samples, immunohistochemical staining was performed to measure the expression of transporters in the intratumor microvessels. The results demonstrated that the expression of glucose transporter 1 (GLUT1) and breast cancer-resistance protein (BCRP) were positively correlated to HER2-positive breast cancer but negatively correlated with triple negative breast cancer, indicating that HER2-positive breast cancer maintains BBB integrity [57].

#### 2.1.3. Factors Mediating Trans-Endothelial Migration in Breast Cancer Brain Metastasis

Following the disruption or remodeling of the inter-endothelial junctions, metastatic cells transmigrate through the BBB. Three genes have been identified to mediate the transmigration of breast cancer cells across the BBB: the α2, 6-sialyltransferase (ST6GALNAC5), heparin-binding epidermal growth factor-like growth factor (HB-EGFHB-EGF) and cyclooxygenase-2 (COX2). ST6GALNAC5has been shown to facilitate the adhesion of breast cancer cells to the BE and induce transmigration across the BBB through cell-surface sialylation. The HB-EGF, a ligand for epidermal growth factor receptor (EGFR), has been shown to enhance breast cancer cells’ invasiveness to the brain [58]. Finally, COX2, an enzyme known to synthesize prostaglandins, appears to induce the expression of matrix metalloproteinase-1 (MMP1) in brain metastatic breast cancer cells, therefore inducing permeability of the BBB [43]. In vitro experiments have shown that COX2 increases TEM in brain metastatic cell lines compared to their parental lines, while inhibiting COX2 expression associated with reduced TEM due to inhibition of MMP1 [58].

Interestingly, the immune system has also been shown to play a role in TEM. Indeed, T lymphocytes were shown to promote BM in estrogen-negative breast cancer through the upregulation of guanylate-binding protein 1 (GBP1), which enhances the ability of breast cancer cells to cross the BBB [59]. Semaphorin 4D (Sema4D; also known as CD100) is another mediator of BM shown to promote the transmigration of circulating tumor cells across the BBB [60]. Indeed, besides the biological role of Sema4D in CNS and the immune system, accumulating evidence supports its association with cancer progression, metastasis, and its ability to disrupt the integrity of BBB in CNS diseases through its interaction with the proangiogenic, high affinity receptor, Plexin-B1 [61,62]. Interestingly, disrupting the interaction of Sema4D with Plexin-B1 receptor has been shown to hinder the transmigration of cancer cells in an in vitro experimental model of the BBB [60].

BM in HER2-positive breast cancer was shown to be mediated by several ligands such as heregulin (HRG) and EGF. Exogenous HRG and MMP proteolytic enzymes (MMP2, MMP9) were shown to enhance the adhesion and TEM across BE in HER2/3-expressing luminal breast cancer cell lines (MDA-MB-361, MCF-7, SKBr3). In HRG-rich brain microenvironment, combined blockade of HER2 and HER3 via the available monoclonal antibodies (MABs) has been shown to abrogate TEM. The results suggest the potential effect of HRG-HER3-HER2 signals in mediating extravasation in breast cancer BM [63]. Moreover, HER-2 expressing MDA-MB-231BR perform TEM to a higher extent in an EGF-rich environment [64].

Connexins (Cx) are proteins which form gap junction channels and maintain intercellular communication [65]. Several reports suggest that metastatic breast cancer cells require Cx43 to extravasate, as knocking out Cx43 in breast cancer cells significantly decreases the extravasation ability. Moreover, activation of the metastatic transcription factor twist in breast cancer cell lines induces CX43 expression, which in turn increases gap junction communication with BECs, mediating rapid extravasation of breast cancer cells [66].

Syndecan-1 (Sdcs-1) is a heparan sulfate proteoglycan (HSPG) present on cell surfaces and acts as a co-receptor to multiple biological factors, including cytokines, matrix proteins, growth factors, and angiogenic factors. Syndecans are often found to be dysregulated in many types of cancer and their overexpression have been detected in ER-negative breast cancer cells (HER2-positive and triple negative breast cancer) but not in ER-positive as Sdc-1 expression is suppressed by ER. The proteoglycan Sdcs-1 facilitates transmigration of breast cancer cells across the BBB by mediating the release of cytokines from tumor cells [67,68,69].

#### 2.1.4. Cytokines Mediating Extravasation in Breast Cancer Brain Metastasis

Cytokines including chemokines, tumor necrosis factors, interferons, and interleukins are soluble factors that regulate cellular migration and mediate the infiltration of cells in specific tissues [70]. Emerging evidence demonstrates the effect of chemokines in the development of cancer and metastasis [71]. In vitro studies revealed that the chemokine (C-X-C motif) ligand CX3CL1 and CXCL13 levels were elevated in the serum of breast cancer patients with brain metastases, indicating that the presence of these chemokines may disrupt the integrity of the BBB and thus are implicated in the process of BM [72]. The chemokine signaling pathway CXCR4/stromal cell-derived factor 1 (SDF-1) have been demonstrated to trigger the phosphatidylinositol 3-kinase (PI3K)/protein kinase B (AKT) pathway, which was linked to breast cancer cells TEM, enhancing vascular permeability. The use of an inhibitor to block this signaling pathway merits further research as it opens new insights in targeting BM [73]. In addition, inhibiting PI3K/AKT or Rac signaling pathways have been shown to prevent the BM of breast cancer cells through reducing adhesion and subsequent transmigration [74].

#### 2.1.5. Endothelial-to-Mesenchymal Transition (EndMT) Mediating Extravasation in Breast Cancer Brain Metastasis

Endothelial-to-mesenchymal transition (EndMT) is a dynamic, complex biological process in which endothelial cells differentiate into and adopt a mesenchymal phenotype through a series of extensive transcriptional reprogramming and molecular events. EndMT has been shown as a crucial step that drives breast cancer cells metastatic extravasation into the brain. Transforming growth factor beta-1 (TGFβ-1) is a cytokine that exerts pleiotropic effects on tumor progression, angiogenesis, and other biological processes. TGFβ-1 expression by cancer cells has been reported and linked to EndMT. During this transitional process, BECs downregulate tight and adhesion junctions (VE-cadherin, occludin, and claudin-5), enhance the expression of mesenchymal and fibroblastic proteins (N-cadherin, integrin β1, and fibronectin), and differentiate to a myofibroblast phenotype. This process is mediated through the expression of fibronectin and SMA (α-smooth muscle actin) that contributes to endothelium contractility and downregulation of VE-cadherin in BECs [75]. Interestingly, EndMT might be partial in cancer, particularly in metastasis. Indeed, intermediate stages of differentiation in tumor-derived ECs have been identified where the ECs display heterogeneity in their capacity to undergo EndMT [76]. Specifically, EndMT driven by a transforming growth factor (TGF-β) was shown to produce different EC phenotypes with different functions that could underline the plasticity and heterogeneity of the tumor vasculature [77].

**Figure 1:**
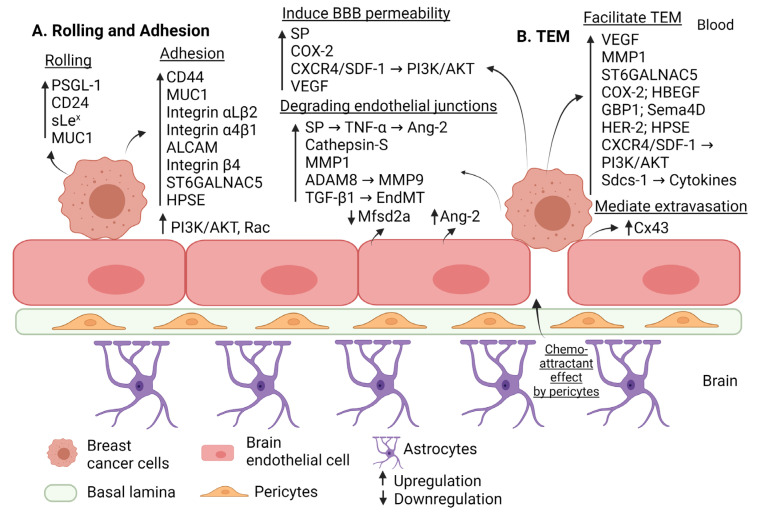
Schematic representation of extravasation steps in breast cancer brain metastasis. (**A**) Rolling and firm adhesion: Rolling and firm adhesion are the first two steps occurring during extravasation, in which ligands expressed on breast cancer cells interact with respective molecules expressed on BECs. During rolling, breast cancer cells express ligands, including PSGL-1, CD24, sLex, and MUC1 that interact with endothelial E-selectin. During adhesion, additional ligands expressed on breast cancer cells, including CD44, MUC1, LFA-1 (Integrin αLβ2), VLA-4 (Integrin α4β1), ALCAM, and Integrin β4, interact with adhesion molecules expressed on the BEC, including VCAM-1 and ICAM-1 to allow the firm adhesion of breast cancer cells on the BEC [28,29]. Mechanistically, through the inflammation and upregulation of PI3K/AKT, Rac signaling pathways have been shown to induce expression of adhesion molecules and promote the adhesion of breast cancer cells on the BE [74]. (**B**) Trans-endothelial migration: Enhanced permeability of BBB in breast cancer BM is mediated by upregulation of inflammatory mediators, including the COX2 and MMPs, leading to the disruption of inter-endothelial junctions, including (ZO-1 and claudin-5) [43]. Upregulation of CXCR4/SDF1 signaling pathway induces vascular permeability and TEM through activating PI3K/AKT and calcium signaling [73]. Upregulation of VEGF increases the permeability of BBB through redistributing actin fiber and disrupting VE-cadherin, thus promoting TEM. Cathepsin-S induces proteolytic cleavage of JAM-B, ADAM8 upregulates MMP9, and EndMT decreases VE-cadherin levels. Other mediators of breast cancer cells TEM include VEGF, MMP1, ST6GALNAC5, COX2, HB-EGF, GBP1, Sema4D, HER2, HPSE, CXCR4/SDF1, PI3K/AKT, Sdcs-1, and Cx43. Pericytes exert a chemoattractant effect on breast cancer cells to promote TEM [38]. Photo created with BioRender.com.

A schematic representation of the molecular mechanisms mediating the extravasation of breast cancer cells is shown in Figure 1. Table 1 summarizes the molecular factors mediating extravasation of breast cancer cells.

#### 2.1.6. MicroRNA Mediating Extravasation in Breast Cancer Brain Metastasis

MicroRNAs (miRs) are a class of small single-stranded, non-protein coding RNA molecules, consisting of around 19–23 nucleotides, and act as post-transcriptional regulators. They modulate gene expression through imperfectly binding to 3′untranslated region (3′UTR) of the target mRNA, suppressing their translation and degrading them [78]. MiR dysregulation is associated with a variety of human diseases, including cancer and their metastatic processes, establishing them as oncogenes or tumor suppressors [79,80]. Accumulating evidence has revealed the role of miRs in BM cascade, including the extravasation process across the BBB [81]. In addition, miRs were shown to play a role in the impairment of the inter-endothelial protein junctions and affect the permeability of the BBB [82,83].

MiR-509 expression levels in brain metastatic lesions are lower compared to the expression levels in primary breast tumors. Researchers discovered that miR-509 suppresses BM by negatively targeting two genes, TNF- and RhoC-induced MMP9, which limit BBB permeability and TEM, respectively. Moreover, an in vivo model confirmed the ability of miR-509 to suppress BM [84]. Additionally, the expression level of miR-101-3p is downregulated in metastatic breast cancer cells. Loss of miR-101-3p upregulates COX2/MMP1 signaling pathway, which contributes to the degradation of BE protein junctions (VE-cadherin and claudin-5), thus disrupting the integrity of the BBB [85]. A synergistic effect was also revealed through combining miR-101-3p and miR-26b-5p, in which their restoration inhibited COX2/MMP1 and suppressed TEM [86].

Moreover, the loss of another microRNA, miR-202-3p, was observed in brain metastatic breast cancer compared to the primary breast tumor. Downregulating miR-202-3p enhanced the transmigration ability of breast cancer cells across the BE by mediating the expression of MMP1, which in turn degraded inter-endothelial protein junctions, ZO-1, claudin-5 and β-catenin. Restoring miR-202-3p expression had an anti-metastatic effect by preserving the integrity of BBB [87]. Similarly, a study showed that restoring the expression of miR-623 attenuated MMP1 expression that controlled TEM [88].

Additionally, the metastatic breast cancer cells express and release microRNAs, such as miR-181c and miR-105, that disrupt the integrity of BE by negatively regulating the expression of ZO-1. An in vivo experiment showed that the upregulation of miR-105 in non-metastatic breast cancer cells promotes metastasis and vascular permeability in remote organs (lung, liver, and brain) while inhibiting miR-105 in the metastatic breast cancer cells attenuated these metastatic properties. Therefore, during the premetastatic stage of breast cancer, an elevated level of miR-105 can be detected in the circulation, suggesting it as a powerful diagnostic biomarker for BM of breast cancer [89].

MiR-181c delocalizes the cytoskeletal protein, actin, by downregulating the expression of its gene, the 3-phosphoinositide-dependent protein kinase-1 gene (PDPK1). PDPK1 phosphorylates cofilin (an actin-binding protein), rendering it inactive, whereas degradation of PDPK1 by miR-181c will activate cofilin, modulating actin dynamics at the level of BBB, and promoting its breakdown [90]. Table 2 summarizes the role of micro-RNAs in mediating the extravasation of breast cancer cells.

### 2.2. Molecular Mechanisms Mediating Extravasation in Melanoma Brain Metastasis

Melanoma is a malignant tumor that arises from pigment-producing cells called melanocytes and is present in the skin, iris, and other parts of the body [91]. Melanoma is the fifth most common malignancy [92] and the third most common cause of BM after lung cancer and breast cancer [93]. More than 50% of advanced melanoma patients develop brain metastasis during the course of the disease [94,95,96,97]. Diagnosis of melanoma brain metastasis is associated with poor prognosis and causes severe neurologic morbidity and death within 3 months if left untreated [98]. Risk factors associated with the development of brain metastasis in melanoma include male sex, high serum lactate dehydrogenase level, high Breslow thickness of primary melanomas, head, or neck as the site of primary disease, and visceral or nodal involvement [99]. The therapeutic options to treat melanoma brain metastases are limited due the presence of the BBB that restricts entry of therapeutic molecules into the brain, as well as the presence of drug efflux pumps [100]. At the molecular level, melanoma cells spread to the brain via hematological dissemination where the extravasation of tumor cells through the BBB represents a critical step in the metastatic cascade.

#### 2.2.1. Factors Mediating Rolling and Firm Adhesion of Melanoma Cells to the Brain Endothelium

Similar to the breast cancer cells, melanoma cell extravasation initially involves their adhesion to BECs, facilitated by integrins. VLA-4, also known as integrin α4β1, is expressed in 92% of human melanoma specimens that metastasize to the brain. Expression of VLA-4 has been found to mediate the adhesion of melanoma cell lines through the interaction with VCAM-1 expressed on the BEC. In contrast, inhibition of VLA-4 abolishes melanoma cell adhesion and intercalation to the BBB, preserving barrier integrity. VLA-4 expression could therefore be explored as a therapeutic intervention to prevent melanoma-BM. Other studies showed that inhibition of PI3K/AKT or Rac signaling pathways in melanoma cell lines prevent tumor cell adhesion and migration through the BE and inhibit BM. Similar to breast cancer, brain pericytes were shown to play and important role in brain metastasis and tumor growth of melanoma cells in the brain [101]. In the extravasation step, pericytes secrete high amounts of extracellular matrix proteins and enhance the adhesion of both melanoma cells to the brain endothelium [38]. However, more studies are needed to better characterize the role of pericytes in the extravasation of melanoma cells.

#### 2.2.2. Factors Affecting the BBB Permeability in Melanoma Brain Metastasis

Through in vitro studies, melanoma cell lines were found to migrate across the BBB under the influence of MMP expression, particularly MMP2 and MMP9, directly affecting BBB permeability and resulting in extravasation. Upon the signal transducer and activator of transcription 3 (STAT3) activation, expression of MMP2 was observed and STAT3 activity was increased in brain metastatic melanoma cells compared to levels in primary melanoma cells, indicating the significant contribution of STAT3 activity in the extravasation process of melanoma BM [102]. Data from the same study also suggest that other MMPs expression (MMP7, 10, 11, 13) may be associated with the BM of melanoma cells [103].

The small acidic EF-hand calcium-binding protein (S100A4) is another factor involved in tumor progression in several types of cancer. Melanoma cell lines express S100A4 in the extracellular region. It autocrinely stimulates the prometastatic activation of melanoma cells by interacting with the receptor for advanced glycation end product (RAGE). Overexpression of S100A4 was also associated with RAGE upregulation, indicating a role for S100A4/RAGE signaling in metastatic activation of melanoma [104]. In addition, S100A4 functions as a paracrine mediator of melanoma BM by interacting with endothelial RAGE. It compromises BEC integrity by degrading occludin and her VE-cadherin at the inter-endothelial junctions and subsequently promotes melanoma cell migration. Furthermore, in vivo experiments have shown that mice injected with melanoma cells and expressing S100A4 or RAGE lead to the formation of brain metastases [105].

Melanoma cells adhere to the BE and migrate through the BBB using the paracellular pathway. During this TEM, melanoma cells release proteolytic enzymes (serine gelatinolytic proteases, including seprase) that degrade basement membrane components. Thus, inhibition of serine proteases was found to significantly reduce BBB extravasation of melanoma cells. Moreover melanoma cells in contact with BECs disrupt the integrity of the BBB by degrading the tight junctional proteins (claudin-5 and ZO-1) [106]. Furthermore, the protease enzymes seprase and the urokinase plasminogen activator receptor (uPAR) expressed in melanoma cells co-localize to form supramolecular structures and provide potent pericellular proteolysis of the ECM required for metastasis. This formation of membrane complex is dependent on cytoskeleton and β1 integrin [107]. Heparanase (HPSE-1), as discussed earlier, is an endoglycosidase that degrades ECM and basement membranes of the BBB to facilitate tumor progression and metastasis to the brain. HPSE expression is increased in brain metastatic melanoma cells, and experiments using an in vivo brain model show that HPSE-1 expression increases melanoma cell invasion into the brain [108]. The expression of HPSE can be suppressed by miR-155, which can block melanoma cells adhesion, invasion, and metastasis, and the expression of chemokines such as interleukin-8 (IL8) and CXCL1 [109].

#### 2.2.3. Factors Mediating Trans-Endothelial Migration in Melanoma Brain Metastasis

Pleckstrin homology domain-containing family A member 5 (PLEKHA5) is an adaptor protein that must associate with other molecules to facilitate its function. It is known to have a phosphoinositide binding specificity, indicating that PLEKHA5 exerts its effect on BM by mediating the PI3K/AKT signaling pathway. Jilaveanu et al. demonstrated that the expression of PLEKHA5 was significantly upregulated in melanoma BM patient samples and a brain metastatic melanoma cell line (A375Br). Furthermore, silencing PLEKHA5 expression in an experiment in vitro decreased the transmigration and invasion of melanoma cells across the BBB [110].

Plasminogen is another factor that has been linked to mediating TEM. It is activated to plasmin, a vital serine protease enzyme involved in the fibrinolytic system, where it either directly or indirectly degrades the ECM. It has been reported that the absence of plasminogen and plasmin inhibitor in vitro showed reduced TEM of melanoma cells. Furthermore, both the wild-type mice treated with plasmin inhibitors and plasminogen-knockout mice had smaller metastatic tumor sizes and longer survival rates than untreated wild-type mice. The results indicate the crucial role of plasmin in promoting the migration of melanoma cells across the BBB [111].

Melanotransferrin (MTf) is one of the antigens expressed on the membrane surface of melanoma cells. In vitro studies confirm that melanoma cells expressing MTf can transmigrate cross the BBB. Since MTf can activate plasminogen to generate plasmin [112,113], the involvement of plasmin, MMP2, and MMP9 leads to a MTf-dependent transmigration across the BBB. MABs have been reported to effectively attenuate TEM in MTf-positive melanoma cells and efficiently reduce BM formation in MTf-positive melanoma cells [114].

#### 2.2.4. Cytokines Mediating Extravasation in Melanoma Brain Metastasis

The signaling between melanoma cells and astrocytes potentiates the metastasis of melanoma cells, particularly due to the pro-inflammatory environment generated by the pre-metastatic mediator interleukin-23 (IL-23). It has been revealed that IL-23, a pro-inflammatory cytokine produced by astrocytes, was sufficient to accelerate brain metastatic melanoma cells’ TEM. IL-23 generated by astrocytes was found to increase MMP2 secretion in brain metastatic melanoma cells, whereas inhibiting MMP2 expression decreased IL-23-induced migration, implying a link between the two [115]. The role for astrocytes has also been demonstrated in promoting melanoma brain metastases. Astrocytes were shown to be activated by melanoma-secreted factors and upregulated the CXCL10 expression, which subsequently drove melanoma cells migration toward astrocytes. In addition, chemokine receptor CXCR3, the CXCL10 receptor, was shown to be overexpressed in brain metastatic melanoma cells, and silencing either CXCR3 or CXCL10 can efficiently reduce migration toward astrocytes [116]. Furthermore, the chemokine receptor CCR4 is overexpressed in samples of brain metastatic melanoma cells as compared to primary tumor cells. CC chemokine ligand CCL17, a CCR4 ligand, was discovered to be released by BECs, astrocytes, and microglia, with their interaction driving melanoma cell migration toward the brain. Specifically, the CCR4/CCL17 axis promotes melanoma cells to migrate toward astrocytes and TEM via the BBB. Taking this into account, an in vivo model revealed that blocking CCR4 using an antagonist molecule reduces tumorigenicity and brain metastases [117].

#### 2.2.5. EndMT Mediating Extravasation in Melanoma Brain Metastasis

**Figure 2:**
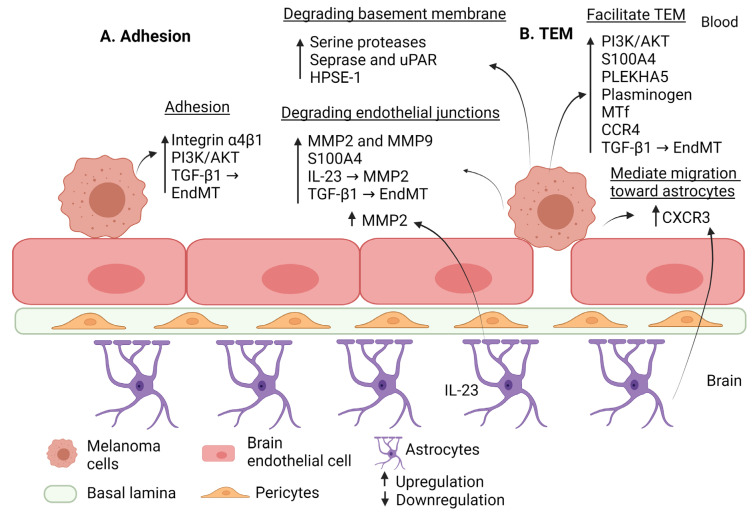
Schematic representation of extravasation steps in melanoma brain metastasis. (**A**) Rolling and firm adhesion: During adhesion to BE, melanoma cells express VLA-4(Integrin α4β1), TGF-β1 (activate EndMT), and upregulate PI3K/AKT signaling pathway that enhance the adhesion of melanoma cells [74,75]. (**B**) Trans-endothelial migration: Disruption of endothelial junctions allows the TEM of melanoma cells. Melanoma cells release MMP2, MMP9, S100A4, and TGF-β1 that stimulates EndMT, which in turn reduces the expression of claudin-5 and VE-cadherin. Transmigrated melanoma cells trigger astrocytes to release IL-23, that in turn upregulates MMP2 in melanoma cells and facilitates TEM. Melanoma cells also release proteolytic enzymes to degrade a basement membrane, including serine proteases, seprase-uPAR complex, and HPSE-1 [109]. Other TEM mediators include PI3K/AKT, S100A4, PLEKHA5, plasminogen, MTf, CCR4, and TGF-β1 that activate EndMT, while CXCL10 secreted by activated astrocytes attracts the migration of melanoma cells toward astrocytes through the secreted CXCR3 [105,110,117,118]. Photo created with BioRender.com.

As previously discussed in breast cancer BM, EndMT appears to be one of the potential mechanisms by which metastatic extravasation takes place. Similarly, researchers found that melanoma cell lines cultured in activated conditioned media induce this transitional process (a TGF-β-dependent manner) in the BEC, as was demonstrated by a reduced expression of claudin-5 and VE-cadherin, and increased expression of fibronectin and SMA. Furthermore, data of this study showed enhanced adhesion and transmigration ability of melanoma cells across BE [75]. A schematic representation of the molecular mechanisms mediating extravasation of melanoma cells is shown in Figure 2. Table 3 summarizes the molecular factors mediating extravasation of melanoma cells.

#### 2.2.6. MicroRNA Mediating Extravasation in Melanoma Brain Metastasis

The role of micro-RNA in melanoma metastasis has been investigated and reviewed elsewhere [120]. However, the role of microRNAs in the extravasation of melanoma cells through the BBB remains largely unexplored. For instance, a miRNA-based signature detected in primary melanoma tissue that predicts the development of brain metastasis has been explored. A retrospective, cohort-based study analyzing genome-wide and targeted microRNA expression profiling of primary melanoma tumors of three-patient cohorts was performed. As a result, a 4-microRNA (miR-150-5p, miR-15b-5p, miR-16-5p, and miR-374b-3p) prognostic signature was shown to distinguish primary melanomas that metastasized to the brain from non-recurrent and non-brain metastatic primary tumors [121]. However, the role of these micro-RNAs in the extravasation step remains unexplored. Similarly, microRNA-146a was shown to suppress melanoma metastatic activity in brain metastasis [122], while miR-124a, a miRNA that is highly expressed in the brain was shown as a suppressor of melanoma metastasis in vivo, particularly of brain metastasis. A significant fraction of melanomas that metastasized to the brain was found to carry a decoy of miR-124a [123]. MicroRNA-23a was shown to inhibit melanoma cell proliferation, migration, and invasion in mice through a negative feedback regulation of sdcbp and the mitogen-activated protein kinase (MAPK)/extracellular signal-regulated kinase (ERK) signaling pathway [124]. MicroRNA-9 was shown to suppress the growth, migration, and invasion of malignant melanoma cells via targeting neuropilin-1 (NRP1) [125]. However, the role of these micro-RNAs in extravasation remains to be determined. In this regard, it is important to highlight the dual role that some micro-RNAs can play in the tumorigenesis process. For instance, a dual role of miR-155 has been reported in melanoma progression. Indeed, while a decreased expression of miR-155 was found in later-stage melanoma and cases with brain metastasis, upregulation of miR-155 has been reported in primary and metastatic melanoma samples. miR-155 expression was associated with favorable outcomes in some studies, while others showed an association with metastasis [126]. Therefore, more mechanistic studies are needed to understand the multifunctional role of micro-RNAs in cancer in general, and brain metastasis in particular.

### 2.3. Molecular Mechanisms Mediating Extravasation in Lung Cancer Brain Metastasis

Lung cancer represents the leading cause of cancer-related mortality and incidence worldwide [127]. According to histopathology, lung cancer can be divided into non-small cell lung cancer (NSCLC), which accounts for 85% of lung cancer cases, and small cell lung cancer (SCLC) that accounts for 15%. Among all cancer types, lung cancer has the highest incidence of BM among all types of cancers [128]. Upon diagnosis, around 10–25% of lung cancer patients present with BM and 40–50% of such patients will develop BM during the course of the disease [129].

#### 2.3.1. Factors Mediating Rolling and Firm Adhesion of Lung Cancer Cells to the Brain Endothelium

A growing body of evidence indicates that cell adhesion molecules (CAMs) are associated with tumor progression and metastasis. The aforementioned IgSF, ALCAM, shows a significant increase in NSCLC brain metastases compared to primary tumors. In vitro experiments showed that deleting ALCAM expression through CRISPR/Cas9 technology reduces tumor cell adhesion to the BE, subsequently decreasing the ability of tumor cells to extravasate through the BBB. Moreover, mice injected with ALCAM-knockdown cells resulted in fewer tumor dissemination to the brain with a significant reduction in both the size and number of BM [130].

Other molecules known to play a role in adhesion are the Lewisx and sLex, also known as fucosylated carbohydrate epitopes CD15 and sialyated CD15s, respectively. Expression levels of CD15 and CD15s epitopes are highest in metastatic NSCLC cells, and the interaction of these epitopes with their E-selectin receptor (CD62E) expressed on BE mediates the adhesion of NSCLC cells to BE. The role of CD15 and CD15s in promoting tumor cell adhesion and disruption of BE can be further validated through the results reported within in vitro studies. These experiments inhibited the expression of CD15 and CD15s by targeting their encoding genes fucosyltransferases 4,7 (FUT4 and FUT7), respectively, which thus yielded in a reduced cancer cells adhesion and preserved integrity of BBB. On the other hand, overexpressing CD15 or CD15s in non-metastatic cell lines led to their conversion to “metastatic-like” in terms of disrupting the integrity of BBB [131,132,133].

Moreover, expression levels of ADAM9 is significantly elevated in brain metastatic NSCLC cells; its overexpression in NSCLC cell lines resulted in increased adhesion to brain tissue through the upregulation of integrin α3β1. In addition, in an in vivo model where mice were injected with ADAM9-overexpressing NSCLC cells, BM was observed [134].

#### 2.3.2. Factors Affecting the BBB Permeability in Lung Cancer Brain Metastasis

Gene analyses have detected multiple genes and pathways that are involved in the function of tight junctions, including genes related to adhesion, Janus Kinase (JAK)-STAT, PI3K, and CAMs pathways [135]. Moreover, experiments showed that upregulating claudin-5 junctional protein in BBB mimicking human brain vascular endothelial (hCMEC/D3) cells attenuates paracellular permeability to lung cancer cells, reduces its migration, and increases its proliferation, whereas knockdown experiments yield opposite findings. Permeability of BBB can also be promoted due to the diminished expression of Mfsd2a that transport the essential fatty acids, such as DHA, as discussed earlier in breast cancer BM [54].

Adenosine A2A receptors are highly expressed in the brain and hence regulate multiple biological functions. Previous evidence has reported that the stimulation of adenosine A2A receptors is involved in cancer progression and metastasis. Contrary to these results, a study revealed that the activation of adenosine A2A receptors inhibits lung cancer BM by interfering with SDF-1/CXCR4 signaling pathway involved in promoting cancer metastasis, and through upregulating the expression level of tight junction proteins; claudin-5, occludin, and ZO-1, thereby preserving the integrity of BBB [136].

Aldo-keto reductase family 1 B10 (AKR1B10) is a multifunctional protein involved in the progression of many types of cancers, high expression levels of which facilitate extravasation of lung cancer cells (NSCLC) across the BBB. The extravasation process has been linked with its role in degrading junctional proteins (ZO-1 and VE- cadherin) to promote TEM of NSCLC cells. Moreover, it has been shown to upregulate MMP2 and MMP9 through the MEK/ERK signaling pathway, which was validated through its silencing. This suppressed the TEM of lung cancer cells in an MMP-dependent manner and inhibited the BM of lung cancer in both the in vivo and in vitro experimental models [137].

#### 2.3.3. Factors Mediating Trans-Endothelial Migration in Lung Cancer Brain Metastasis

SCLC patients with BM have higher levels of annexin A1 as compared to SCLC patients without BM, as was observed through studies on patient samples and SCLC cell line, which showed overexpression of annexin A1. Annexin A1 belongs to annexin super-family proteins that is believed to have a role in tumor development. The results demonstrate that annexin A1 is not only overexpressed in SCLC cell line but is also secreted into extracellular space through its interaction with the BE. The upregulated annexin A1 levels in SCLC cell line promoted their adhesion to BE subsequently facilitating their TEM. Moreover, silencing annexin A1 expression in SCLC cells inhibited their TEM in the in vitro model and attenuated BM formation in the in vivo mice model [138].

Moreover, in SCLC, the BE Rho/ROCK signaling pathway is required for TEM of tumor cells across BBB. In vitro experiments demonstrate that transmigration of tumor cells results in the redistribution of tight junction proteins (occludin, claudin-5 and ZO-1), while inhibiting endothelial the Rho/ROCK signaling pathway prevents this TEM of SCLC and maintains the assembly of tight junction proteins. Moreover, activation of Rho/ROCK signaling pathway facilitates TEM of SCLC cells through altering actin cytoskeleton in BECs [139].

Placental growth factor (PLGF), a member of the VEGF subfamily, is involved in cancer progression. Serum levels of PLGF were significantly higher in SCLC patients with BM compared to non-metastatic and normal specimens. This is because PLGF secreted from SCLC promotes TEM across BE and disrupts tight junction integrity (occludin and ZO-1). The resultant disassembly of tight junction proteins requires PLGF to bind to its receptor on BE (VEGF receptor-1; VEGFR-1) in the process of TEM, subsequently triggering the activation of Rho/Rho-associated protein kinase (ROCK) signaling pathway and its downstream signal ERK1/2 in the BE [140].

Visfatin is an adipocyte hormone expressed in several tissues, particularly in visceral fat tissues, and contribute to multiple biological functions associated with several types of malignancies [141]. Visfatin levels are additionally elevated in SCLC patients’ serums who developed BM, which act as a pro-inflammatory cytokine. During its interaction with BECs, visfatin is upregulated in SCLC cell lines, which in turn induces the expression of chemokine ligand 2 (CCL2) in SCLC cell lines, promoting their TEM. Furthermore, study findings also indicate that visfatin-induced CCL2 expression in SCLC cells is mediated by PI3K/Akt signaling pathway [142]. Table 4 lists the factors mediating TEM in lung cancer BM.

#### 2.3.4. Cytokines Mediating Extravasation in Lung Cancer Brain Metastasis

Using a syngeneic orthotopic cerebral metastasis model in mice, Zhang et al. showed that CX3CR1 deficiency resulted in a lower number of extravasated tumor cells, although the progression of the extravasated cells into micro-metastases was more efficient. However, the study found that unspecific inhibition of CX3CR1 might not be a suitable therapeutic option to prevent dissemination of lung cancer cells to the brain [143].

In an in vitro model of brain endothelial cells hCMEC/D3, TNFα has been shown to enhance E-selectin (CD62E) expression and therefore the adhesion of non-small cell lung cancer cells to brain endothelium in lung-brain metastasis [131].

In an in vivo experimental model using human lung cancer-derived (HARA-B) cells in nude mice, a significant increase in glial fibrillary acidic protein (GFAP)-positive astrocytes around metastatic lesions was detected. The study found that astrocytes were activated by tumor cell-oriented factors, including macrophage migration inhibitory factor (MIF), IL-8, and plasminogen activator inhibitor-1 (PAI-1). Activated astrocytes produced IL-6, TNF-α, and IL-1β, which in turn promoted tumor cell proliferation [144]. However, the role of these cytokines in the extravasation step remains to be determined.

Similarly, activation of CXCR4 by its ligand CXCL12 has been shown to play an important role in the process of NSCLC metastatic spread to the brain; however, the role of this axis in the extravasation step remains to be fully characterized [145].

#### 2.3.5. EndMT Mediating Extravasation in Lung Cancer Brain Metastasis

**Figure 3:**
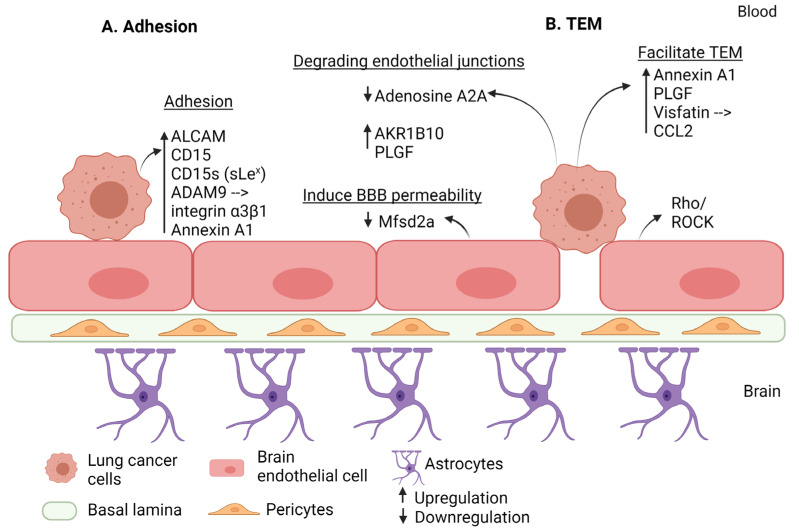
Schematic representation of extravasation steps in lung cancer brain metastasis. (**A**) Rolling and firm adhesion: During adhesion to BE, lung cancer cells express ALCAM, CD15, CD15s (sLex), annexin A1 and ADAM9 that upregulate integrin α3β1 [35,130,134]. (**B**) Trans-endothelial migration: During TEM, BECs downregulate the expression of Mfsd2a to induce permeability of BBB. Downregulation of adenosine A2A receptor in lung cancer cells activate SDF-1/CXCR4 signaling pathway and inhibits the expression of claudin-5, occludin, and ZO-1 [136]. Upregulation of AKR1B10, degrades (ZO-1 and VE- cadherin) and increases the levels of MMP2 and MMP9 [137]. BE Rho/ROCK signaling facilitates TEM with subsequent disruption of (occludin, claudin-5, and ZO-1) [139]. PLGF disrupts occludin and ZO-1 through interacting with its receptor, VEGFR-1 [140]. Visfatin upregulates CCL2 to promote TEM [142]. Photo created with BioRender.com.

Studies have investigated the role of EndMT in the metastasis of lung cancer cells, but the role of EndMT in the brain extravasation step remains less explored. Endothelial cells co-cultured with lung cancer cells were shown to acquire a mesenchymal phenotype with increased vimentin, alpha smooth muscle actin and Smad2/3, and reduced VE-cadherin. These changes were prevented in the presence of [Zn(PipNONO)Cl], suggesting [Zn(PipNONO)Cl] as a promising therapeutic tool to control tumor growth and metastasis [146]. In another study, alpha-1 antitrypsin was shown to induce EndMT in lung cancer cells, suggesting alpha-1 antitrypsin as a factor associated with tumor metastasis in lung carcinoma [147]. However, more studies are needed to understand the role of EndMT in mediating the extravasation of lung cancer cells through the brain endothelium (Figure 3 and Table 4).

**Table 4:** Factors that mediate extravasation during brain metastasis of lung cancer.

Step of Extravasation	Factor	Effect	Regulation in BM	Reference
Adhesion	ALCAM	Increases tumor cell adhesion to BE.	Upregulation	[130]
	CD15	Interacts with E-selectin (CD62E) to mediate adhesion of tumor cells to BE and disrupts the BBB.	Upregulation	[133]
	CD15s (sLe^x^)	Interacts with E-selectin (CD62E) to mediate adhesion of tumor cells to BE and disrupts the BBB.	Upregulation	[133]
	ADAM9	Increases adhesion to brain tissues through upregulating integrin α3β1.	Upregulation	[134]
	Annexin A1	Promotes tumor adhesion to BE.	Upregulation	[138]
Alteration in the BBB permeability	Mfsd2a	Its downregulation in BE will promote permeability of BBB.	Downregulation	[54]
	Claudin-5	Its downregulation will promote permeability of BBB.	Downregulation	[135]
	Adenosine A2A receptor	Its downregulation will activate SDF-1/CXCR4 signaling pathway and inhibit the expression of claudin-5, occludin, and ZO-1.	Downregulation	[136]
	AKR1B10	Degrades junctional proteins level (ZO-1 and VE- cadherin) to promote TEM. Upregulates MMP2 and MMP9 via MEK/ERK signaling pathway.	Upregulation	[137]
	Rho/ROCK	BE Rho/ROCK signaling pathway facilitate TEM with subsequent disruption of tight junction proteins (occludin, claudin-5, and ZO-1).	Upregulation	[139]
	PLGF	Disassembly of tight junction proteins (occludin and ZO-1) via subsequent activation of VEGFR-1, Rho/ROCK, and ERK signaling pathways.	Upregulation	[140]
Trans-endothelial migration	AKR1B10	Degrades junctional proteins level (ZO-1 and VE- cadherin) to promote TEM.	Upregulation	[137]
	annexin A1	Promotes tumor adhesion to BE and thus facilitating TEM.	Upregulation	[138]
	Rho/ROCK	BE Rho/ROCK signaling pathway facilitated TEM with subsequent disruption of tight junction proteins (occludin, claudin-5, and ZO-1).	Upregulation	[139]
	PLGF	Promotes TEM.	Upregulation	[140]
	Visfatin	Upregulates CCL2 via PI3K/Akt signaling pathway, promoting TEM.	Upregulation	[142]
	miR-143-3p	Increases TEM through BBB.	Upregulation	[148]

#### 2.3.6. MicroRNA Mediating Extravasation in Lung Cancer Brain Metastasis

Since the role of microRNA in many types of malignancies has been established, miR-143-3p has been found to be elevated in patients with BM lung cancer. MiR-143-metastatic 3p’s action is mediated by downregulating the expression of vasohibin-1 (VASH1), which has been demonstrated to have an anti-angiogenic function through negatively regulating VEGFA. The in vitro BBB model revealed that miR-143-3p promotes lung cancer BM by increasing TEM of NSCLC cell lines via BBB [148]. Down-regulated microRNA-375 expression is suggested as a predictive biomarker in non-small cell lung cancer brain metastasis [149]. Another study suggested that the expression of miRNA-328 and miRNA-378 in tumor tissues can be used to predict brain metastases in patients with non-small-cell lung cancer, where miRNA-328 might promote brain metastases by regulating the expression of protein kinase Cα [150]. However, similar to melanoma, the role of microRNAs in the extravasation of lung cancer cells through the BBB remains largely unknown. Very few studies have investigated this role. Using endothelial monolayers and mouse models, Wu et al. found that TGF-β1-mediated NSCLC-derived exosomes alter the tight junctions and increase BBB permeability to promote NSCLC brain metastasis via the miRNA-1207-5p/EPB41L5 axis, where the overexpression of lnc-MMP2-2 in brain endothelial cells increased vascular permeability and the inhibition of lnc-MMP2-2 alleviated these effects. In vivo, the authors have found that lnc-MMP2-2 knockdown markedly reduced NSCLC brain metastasis. Mechanistically, lnc-MMP2-2 was found to serve as a microRNA sponge or a competing endogenous RNA for miR-1207-5p and consequently modulated the derepression of EPB41L5 known to promote EndMT, alter tight junctions, and increase the barrier permeability [118].

## 3. Comparison of the Molecular Mechanisms Mediating Extravasation in the Three Types of Cancer: Breast Cancer, Melanoma, and Lung Cancer

**Figure 4:**
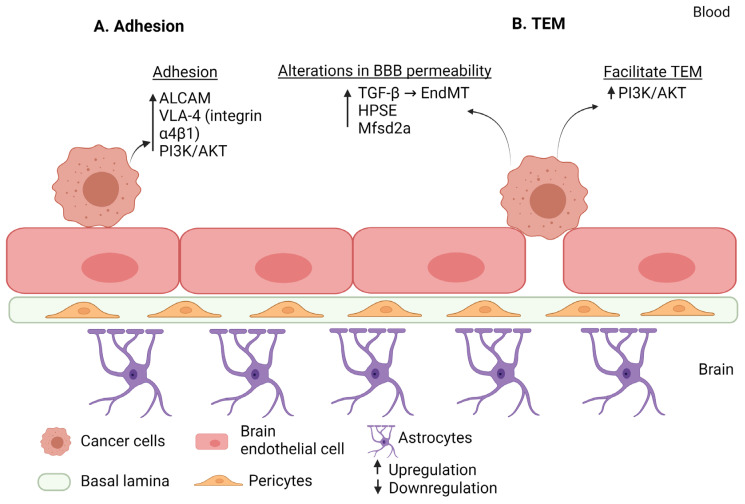
Schematic representation of common mediators of extravasation in the different types of tumors. (**A**) Adhesion: ALCAM is a common adhesion molecule used by both breast and lung cancer cells, while VLA-4 and the PI3K/AKT signaling are used by breast cancer and melanoma cells to promote adhesion [28,130]. (**B**) Trans-endothelial migration: Breast cancer and melanoma cells enhance the BBB permeability through upregulation of HPSE and TGF-β1 and activation of EndMT, while Mfsd2a is a common mediator used by breast and lung cancer cells to induce alterations in the endothelial barrier [53,108]. TEM is commonly mediated by PI3K/AKT in breast cancer and melanoma cells [74]. Photo created with BioRender.com.

Clarification of the molecular mechanisms underlying BM of cancer will provide a basis for the treatment or prevention of such a manifestation. In this review, we focused on the molecular mechanisms mediating the extravasation process, a key step in the metastatic cascade in three different types of cancer most likely to metastasize to the brain: breast cancer, melanoma, and lung cancer. Commonalities in the molecular mechanisms driving extravasation and that may link these different tumors are discussed below and represented in Figure 4 and Table 5.

An initial step of extravasation is the adhesion of tumor cells to the BEC. Several CAMs such as selectins, integrins, and IgSF facilitate the interaction of different types of cancer cells with the BE [34]. Breast cancer cells express multiple types of CAM, including PSGL-1, CD24, sLex, MUC1, CD 44, MUC1, LFA-1 (integrin αLβ2), VLA-4 (integrin α4β1), and ALCAM, whereas melanoma and lung cancer have limited CAM expression [28,29,30,33,34]. However, the overexpression of αv-integrins, particularly αvβ3 and αvβ8 integrins, have been observed in the BM of the three types of cancer [151] which aid in BM by promoting tumor adhesion to brain vasculature [152]. Further studies are required to investigate the role of CAMs in mediating extravasation of cancer cells to the brain, and to test the potential role of CAM inhibitors as therapeutic intervention for BM.

Breast cancer, melanoma, and lung cancer cell lines that expressed factors disrupting tight junction and/or adherens junction proteins display increased BBB permeability, consequently facilitating TEM. Moreover, multiple proteolytic enzymes are involved in BM and numerous studies have revealed the significant role of MMPs in cancer progression and metastasis through BBB disruption. Since certain MMPs are upregulated in the BM of breast cancer, melanoma and lung cancer, an increased interest has been garnered around designing MMP-specific molecular imaging techniques to detect diagnostic biomarkers against BM. Therefore, additional studies could further reveal the role of other MMPs in BM [153]. ADAM8 is another proteolytic enzyme which promotes the TEM of breast cancer cells through upregulating MMP9, while ADAM9 facilitates the adhesion of NSCLC cells through upregulating integrin α3β1 [45,134]. HPSE has been found to be a common BM mediator in both melanoma and breast cancers, facilitating BM either through its endoglycosidase activity or through mechanisms unrelated to its enzymatic activity, respectively [53,108]. Therefore, it is of clinical importance to develop molecules that can efficiently inhibit the activity of these enzymes or prevent their expression levels.

Loss of Mfsd2a, a DHA transporter in breast and lung cancer, is seen as a common mechanism utilized by these two types of cancers that promote BM through disrupting the integrity of BBB, suggesting the importance of restoring DHA levels in the brain as a potential therapeutic target to suppress BM [54].

Moreover, the role of VEGF in cancer progression and metastasis has been studied [154]. Overexpression of VEGF affects the extravasation process during the BM of lung and breast cancer, where it promotes TEM and disrupts BBB integrity. This suggests that VEGF could be a potential therapeutic target for BM, and the identification of this diagnostic biomarker can assist in selecting patients who might respond to the anti-VEGF agents [48,140]. Other soluble factors that are associated with BM are the cytokines, in which its release promotes various steps of metastasis, including the extravasation of breast cancer, melanoma, and lung cancer, thereby indicating the crucial role of chemokines as therapeutic targets in BM [70].

Although most studies on breast cancer BM have been performed on the triple negative breast cancer cell lines [155], it has been demonstrated that BM of HER2-positive breast cancer preserves the integrity of BBB through expressing transporters (GLUT1 and BCRP) in the intratumor microvessels compared to the triple negative breast cancer that shows a negative correlation with these transporter expressions. Therefore, the data indicate that the BM of these two types of breast cancer can differ with regard to the maintenance or disruption of BBB integrity, depending on breast cancer cells affinity to brain tissue. However, additional studies are required with large sample sizes to address this interpretation and to confirm the results [57]. Furthermore, it has been shown that HER3-HER2 dimer facilitates BM in a heregulin-dependent manner. Therefore, targeting the HRG-HER3-HER2 signal in HER2-positive breast cancer can contribute to a promising role in preventing BM [63].

In a study comparing melanoma cells with breast cancer cells, melanoma cells had advanced adhesion to BE, more rapid transmigration, and a higher ability to disrupt inter-endothelial tight junctions than breast cancer cells, which explains the higher brain metastatic propensity of melanoma cells. Inhibition of Rac or PI3K/Akt signaling pathways in melanoma and breast cancer cells reduced their adhesion and transmigration across the BE [74].

The PI3K/Akt signaling pathway is a key regulator pathway that promotes cellular processes, including cell survival, proliferation, and angiogenesis. Accumulated evidence highlights the involvement of PI3K/AKT in BM of breast, melanoma, and lung cancer [8,156,157]. While it has been demonstrated that PI3K/AKT only promotes TEM in lung cancer, it has been found to assist extravasation in breast cancer and melanoma by promoting adhesion as well as the transmigration of tumor cells across the BE [74,142].

Inflammation plays a crucial role in the metastasis of cancer cells. Several studies have investigated the role of cytokines in the brain extravasation step. CX3CL1 and CXCL13 were shown to play an important role in the brain extravasation of breast cancer [72], while the following cytokines/chemokines were involved in the extravasation of melanoma cells (9IL-23 [115], CXCL10 [116], CXCR3 [116], CXCL10 receptor [116], CCR4 [117], CCL17 [117], and CCR4 [117]) and lung cancer cells (TNF-α [131] and CX3CR1 [143]). However, research in this area remains limited and more studies are still needed to fully understand the regulation of extravasation by inflammation and cytokines.

EndMT has been shown as a crucial step that drives cancer cells extravasation into the brain. Interestingly, EndMT has a potential role in mediating metastatic extravasation of breast cancer and melanoma toward the brain. Specifically, TGFβ-1 expression by breast cancer and melanoma cells has been reported and linked to EndMT [75,158]. However, few studies have investigated the role of EndMT in the extravasation of lung cancer cells. More studies are still needed to understand the role of EndMT in mediating the extravasation of cancer cells through the brain endothelium.

A significant amount of evidence explores the role of miRs in mediating cancer metastasis steps; however, the role of microRNAs in extravasation merits more studies. Studies revealed an involvement of a number of microRNAs in the extravasation of breast cancer cells through the BBB, including miR-509, miR-101-3p, miR-26-5b, miR-202-3p, miR-623, miR-105, miR-181c, and miR-1258. Regarding melanoma and lung cancer, little studies have explored the role of microRNA in extravasation. miR-150-5p, miR-15b-5p, miR-16-5p, miR-374b-3p, miR-124a, and miR-155 were shown to be involved in brain metastasis of melanoma while miR-143-3p, microRNA-375, miRNA-328, miRNA-378, and miRNA-1207-5p in brain metastasis of lung cancer. Each type of cancer seems to have a specific micro-RNA signature associated with brain metastasis rather than common micro-RNAs patterns. Finding miRNA signatures to be measured for discriminating between different stages and types of cancer is of utmost importance [159]. In addition, a better understanding of the multifunctional role of micro-RNAs in the metastasis of the different types of cancer is needed. Indeed, several micro-RNAs were shown to exert a tumor-suppressor effect in a type of cancer, while being tumor inducers in another type. For instance, miR-24 is differentially expressed in different type of cancers, with opposite roles as an oncogene or a tumor suppressor [160]. As miRs can serve as a potential biomarker to detect the formation of BM in multiple types of cancer, intense research is needed to better understand the role of miRs in driving BM formation.

In the previous section, we discussed the common mechanisms mediating extravasation in breast cancer, melanoma, and lung cancers. As for the differences, few studies have investigated the unique molecular mechanisms mediating extravasation in a specific type of cancer and not in the other. Extravasation has been better studied in breast cancer compared to the two other type of cancers. However, whether the factors shown to mediate extravasation in breast cancer are also involved in the other types of cancers remains to be determined. This lack of knowledge merits more investigation. We believe that the steps of extravasation are essentially the same for the different types of tumors; the first two steps of the extravasation of tumor cells and leukocytes, rolling and adhesion, seem to have similarities with regard to the mechanisms and receptors involved; however, the molecules involved are different [161]. This is also true for the different subtypes of the same type of cancer. For instance, HER2 has been shown to be involved in the extravasation of HER2+ breast cancer cell, while this receptor is not expressed in triple negative breast cancer cells [57,63]. Although it remains necessary to identify the common mechanism in order to eventually identify common therapeutic targets, it is of utmost importance to unravel the molecular mechanisms specific for each type and subtype of cancer to allow a personalized treatment of patients diagnosed with cancer, a disease characterized by a complex heterogeneity.

## 4. Potential Anti-Brain Metastasis Strategies Targeting the Extravasation Step and Future Research Lines

Current therapeutic options to treat brain metastasis include surgical resection, stereotactic radiosurgery, whole-brain radiation therapy, chemotherapy, and targeted therapy. One challenging problem in treating BM is the inability of certain drugs to cross the BBB and reach the brain. The different current therapeutic treatments and emerging treatment strategies to treat BM have been revised elsewhere [162,163,164,165,166]. However, none of these therapeutical strategies target the steps of extravasation to suppress entry of cancer cells into the brain. In Table 6, and based on the molecular mechanisms discussed above, we summarize the potential molecular targets and therapeutic strategies that could be used to suppress the extravasation step and therefore prevent BM.

However, despite advancements in cancer treatment and in understanding the molecular mechanisms of carcinogenesis, there is still a lack of knowledge with regard to the molecular mechanisms mediating the extravasation of the different types and subtypes of cancers through the BE. Future research should focus on better understanding the molecular mechanisms mediating each step of the extravasation process. The role of microRNAs as well as exosomes and long-noncoding RNAs should be better characterized. In addition, the role and interaction between the different components of the BBB, especially astrocyte and pericytes, need to be better investigated. In summary, future research should focus on unraveling molecular signatures specific for each type of cancer to allow a personalized treatment.

## 5. Conclusions

Despite the advancements in systemic treatments, the incidence of BM is increasing. The molecular mechanisms driving BM, particularly those driving the extravasation of cancer cells through the BBB, remain poorly understood. Treatment modalities toward BM are an urgent medical need that necessitates further understanding and investigations to identify the precise molecular mechanisms mediating it. In this context, more research is needed to better understand these molecular mechanisms, as well as the role of the different components of the barrier, particularly astrocytes and pericytes, the interaction between the different molecules, and the differences and commonalities between the different types of cancers. Future research should focus on unraveling molecular signatures specific for each type of cancer to allow a personalized treatment. This will aid in developing novel and personalized therapeutic interventions for BM.

Finally, beside its multi-step nature, metastasis is a non-random organotropic process. However, the mechanisms that drive organotropism remain a mystery and the genetic and epigenetic predisposition and adaptation mechanisms that govern brain-tropism of metastatic cancer cells are poorly understood and remain among the most important challenges in cancer research. Understanding these molecular mechanisms can prompt the development of new therapies to suppress or prevent brain metastasis in the different types of tumors susceptible to spread to the brain.

## Figures and Tables

**Table 1:** Factors that mediate extravasation during breast cancer brain metastasis.

Step of Extravasation	Factor	Effect	Regulation in BM	Reference
Rolling	PSGL-1 CD24 sLe^x^ MUC1	Interacts with endothelial E-selectin to induce rolling.	Upregulation	[28] [29] [29] [33]
Adhesion	ALCAM	Interacts with endothelial ALCAM to induce adhesion.	Upregulation	[28]
	VLA-4 (integrin α4β1)	Interacts with endothelial VCAM-1 to induce adhesion.	Upregulation	[28]
	CD 44	Interacts with endothelial E-selectin to induce adhesion.	Upregulation	[30]
	Hyaluronan	Interacts with CD44 to induce adhesion, BE disruption and migration of triple negative breast cancer.	Upregulation	[32]
	LFA-1 (integrin αLβ2) MUC1	Interacts with endothelial ICAM-1 to induce adhesion.	Upregulation	[28] [33]
	Integrin β4	Mediates indirect adhesion to BE through inducing VEGF that disrupt (ZO-1, VE-cadherin).	Upregulation	[35]
	Pericytes	Secrete high amounts of extracellular matrix proteins and enhance adhesion.	Activation	[38]
	HPSE	Induces adhesion through driving the expression of integrin β1 by brain metastatic triple negative breast cancer cells and the expression of VCAM-1 in BECs.	Upregulation	[53]
	ST6GALNAC5	Induces adhesion to BE through cell-surface sialylation.	Upregulation	[58]
	PI3K/AKT	Induces adhesion.	Upregulation	[74]
Alteration of the BBB permeability	MMP1	Degrades claudin-5 and occludin.	Upregulation	[43]
	COX2	Increases permeability of BBB through prostaglandin generation and upregulating MMP1.	Upregulation	[43]
	ADAM8	Upregulates MMP9 and shed of PSGL-1 from breast cancer cells.	Upregulation	[45]
	Substance P (SP)	Increases BBB permeability and disrupts ZO-1, claudin-5 (through inducing TNF-a that in turn augment the secretion of Ang-2).	Upregulation	[46]
	Ang-2	Secreted by endothelial cells to cause disruption of ZO-1, claudin-5; partially dependent on VEGF expression.	Upregulation	[47]
	VEGF	Increases permeability of BBB through redistributing actin fiber and disrupting VE-cadherin.	Upregulation	[48]
	Cathepsin S	Proteolytic cleavage of JAM-B.	Upregulation	[49]
	Mfsd2a	Its downregulation in BE will promote permeability of BBB.	Downregulation	[54]
	BCRP	Induces BBB permeability and preserves BBB integrity.	Downregulation	[57]
	Glut1	Preserves BBB integrity.	Upregulation	[57]
	Glut1	Induces BBB permeability.	Downregulation	[57]
	CXCR4/SDF1	Increases vascular permeability through activating PI3K/AKT pathway and calcium signaling.	Upregulation	[73]
	EndMT (TGF-β dependent manner)	Decreases VE-cadherin levels.	Upregulation	[75]
Trans-endothelial Migration	MMP1 VEGF HPSE COX2 HB-EGF ST6GALNAC5 HER2 PI3K/AKT	Increases TEM.	Upregulation	[43] [48] [53] [58] [58] [58] [64] [74]
	GBP1	Triggered by T lymphocytes to induce transmigration.	Upregulation	[59]
	Sema4D	Interacts with its receptor Plexin-B1 to induce transmigration.	Upregulation	[60]
	HRG-HER3-HER2	Induces adhesion and TEM; increases expression of MMP2, MMP9.	Upregulation	[63]
	Cx43	Mediates extravasation.	Upregulation	[66]
	Sdcs-1	Facilitates transmigration through inducing cytokines.	Upregulation	[69]
	CXCR4/SDF1	Induces TEM by activating PI3K/AKT and calcium signaling.	Upregulation	[73]

**Table 2:** MicroRNAs that mediate extravasation in breast cancer brain metastasis.

MiR	Target	Mechanism	Regulation in Breast Cancer BM	Reference
MiR-1258	HPSE	Increases HPSE expression and activity, mediating BM.	Downregulation	[52]
MiR-509	TNF-α and RhoC-induced MMP9	Induces BBB permeability and TEM.	Downregulation	[84]
MiR-101-3p	COX2-MMP1	Degrades BE protein junctions (VE-cadherin and claudin-5).	Downregulation	[85]
MiR-101-3p and miR-26-5b	COX2-MMP1	Degrades BE protein junctions (claudin-5, VE-cadherin, ZO-1, and β-catenin).	Downregulation	[86]
MiR-202-3p	MMP1	Degrades BE protein junction (ZO-1, claudin-5, and β-catenin).	Downregulation	[87]
MiR-623	MMP1	Degrades BE protein junction (VE-cadherin and claudin-5).	Downregulation	[88]
MiR-105	ZO-1	Disrupts BE.	Upregulation	[89]
MiR-181c	PDPK1	Delocalizes actin fiber, inducing the destruction of BBB.	Upregulation	[90]

**Table 3:** Factors that mediate extravasation during brain metastasis of melanoma.

Step of Extravasation	Factor	Effect	Regulation in BM	Reference
Adhesion	Pericytes	Secrete high amounts of extracellular matrix proteins and enhance adhesion.	Activation	[38]
	PI3K/AKT EndMT (TGF-β-dependent manner)	Enhance adhesion and transmigration.	Upregulation	[74] [75]
	VLA-4 (integrin α4β1)	Interacts with endothelial VCAM-1 to facilitate adhesion of melanoma cells to the BBB.	Upregulation	[119]
Alteration in the BBB permeability	EndMT (TGF-β-dependent manner)	Reduces expression of claudin-5 and VE-cadherin.	Upregulation	[75]
	STAT3	Upregulates MMP2 expression and subsequently enhances BM.	Upregulation	[102]
	MMP2 and MMP9	Disrupts BBB.	Upregulation	[103]
	S100A4	Interacts with RAGE to induce degradation of inter-endothelial junctions, occludin, and VE-cadherin.	Upregulation	[105]
	IL-23	Produced by astrocytes to induce TEM; upregulates MMP2 expression in melanoma cells.	Upregulation	[115]
Degradation of the basement membrane	Serine proteases	Degrades basement membrane components, mediating extravasation.	Upregulation	[106]
	Seprase and uPAR	Forms a membrane complex to degrade ECM.	Upregulation	[107]
	HPSE-1	Degrades ECM of BBB.	Upregulation	[108]
Trans-endothelial migration	PI3K/AKT	Induces adhesion and transmigration.	Upregulation	[74]
	EndMT (TGF-β dependent manner)	Enhances adhesion and transmigration.	Upregulation	[75]
	S100A4	Promotes transmigration of melanoma cells.	Upregulation	[105]
	PLEKHA5	Induces transmigration through mediating PI3K/AKT pathway.	Upregulation	[110]
	Plasminogen	Promotes TEM.	Upregulation	[111]
	MTf	Promotes TEM.	Upregulation	[114]
	IL-23	Produced by astrocytes to induce TEM. Upregulates MMP2 expression in melanoma cells.	Upregulation	[115]
	CXCL10	Produced by astrocytes to enhance migration of melanoma cells toward astrocytes via secretion of CXC3R.	Upregulation	[116]
	CCR4	Interaction of CCR4 with its ligand CCL17 promotes TEM of melanoma cells.	Upregulation	[117]

**Table 5:** Common factors mediating brain extravasation during brain metastasis in different types of cancers.

Step of Extravasation	Factor	Commonality	Reference
Adhesion	PI3K/AKT	Breast cancer Melanoma	[74] [74]
	VLA-4 (integrin α4β1)	Breast cancer Melanoma	[28] [119]
	ALCAM	Breast cancer Lung cancer	[28] [130]
Alteration in the BBB permeability	HPSE	Melanoma Breast cancer	[53] [108]
	Mfsd2a	Breast cancer Lung cancer	[54] [54]
	EndMT (TGF-β dependent manner)	Breast cancer Melanoma	[75] [75]
Trans-endothelial migration	PI3K/AKT	Breast cancer Melanoma	[74] [74]

**Table 6:** Potential pre-clinical therapeutic strategies targeting the extravasation step to suppress BM.

Step of Extravasation Targeted	Strategy	Effect	Type of Cancer	Reference
Adhesion	VLA-4 -1 and ALCAM blocking	Reduction of adhesion and extravasation across the BE	Breast cancer	[28]
	Inhibition of VCAM-1, and/or its specific ligand VLA-4/α4β1 integrin	Reduction of invasion and metastasis	Breast cancer	[28]
	CD44 knockdown	Reduction of the pericellular HA coat on cancer cells, ands consequently, cancer cells adhesion and invasion through the BE	Breast	[32]
	Inhibition of PI3K/AKT or Rac signaling pathways	Prevention of tumor cell adhesion and migration through the BE and inhibition of BM	Melanoma	[74]
	Inhibition of VLA-4	Reduction of cell adhesion and preserving barrier integrity	Melanoma	[105]
	Suppression of HPSE by miR-155	Block adhesion, invasion, and metastasis	Melanoma	[109]
	Deleting ALCAM expression through CRISPR/Cas9 technology	Reduction of adhesion and extravasation across the BE	Lung cancer	[130]
	Inhibition of CD15 and CD15s by targeting their encoding genes (FUT4 and FUT7)	Reduction of adhesion and maintenance of BBB integrity	Lung cancer	[131,132,133]
Alteration in the BBB permeability	Knocking down MMP1 expression	Decreased BM	Breast cancer	[44]
	Combination treatment of an Ang-2 inhibitor with a VEGF inhibitor	Preservation of BBB integrity	Breast cancer	[47]
	miR-509	Suppression of BM by targeting TNF- and RhoC-induced MMP9, which limits BBB permeability and TEM	Breast cancer	[84]
	Combinatorial restoration of miR-101-3p and miR-26b-5p	Preserving the BBB integrity and suppression of TEM by inhibition of COX2/MMP1	Breast cancer	[86]
	miR-202-3p miR-623	Preserving the BBB integrity and suppression of TEM by inhibition of MMP1	Breast cancer	[87] [88]
	Inhibition of serine proteases	Reduction of extravasation by preserving BBB integrity	Melanoma	[106]
	Activation of adenosine A2A receptors.	Inhibition of BM by interfering with SDF-1/CXCR4 signaling and preserving the integrity of BBB	Lung cancer	[136]
Trans-endothelial migration	Inhibition of cathepsin S	Reduction of TEM	Breast cancer	[49]
	Inhibition of COX2	Reduction of TEM due to inhibition of MMP1	Breast cancer	[58]
	Disruption of the interaction of Sema4D with Plexin-B1 receptor	Reduction of TEM	Breast cancer	[60]
	In a HRG-rich brain microenvironment, combined blockade of HER2 and HER3 via monoclonal antibodies	Reduction of TEM	Breast cancer	[63]
	Knocking out Cx43	Reduction of TEM	Breast cancer	[66]
	Blocking CXCR4/SDF-1 pathway	Reduction of TEM	Breast cancer	[73]
	Inhibition of PI3K/AKT or Rac signaling pathways	Reduction of adhesion and TEM	Breast cancer	[74]
	Silencing PLEKHA5	Reduction of TEM and invasion	Melanoma	[110]
	Plasmin inhibitor	Reduction of TEM	Melanoma	[111]
	Blocking Mtf	Attenuates TEM and reduced BM formation	Melanoma	[114]
	Silencing CXCR3 or CXCL10	Reduced migration toward astrocytes	Melanoma	[116]
	Blocking CCR4	Reduction of TEM and BM formation	Melanoma	[117]
	Inhibition of MEK/ERK signaling	Suppression of TEM in an MMP-dependent manner	Lung cancer	[137]
	Silencing annexin A1	Inhibition of TEM and attenuation of BM formation	Lung cancer	[138]
	Inhibition of endothelial Rho/ROCK signaling pathway	Inhibition of TEM and maintaining the assembly of tight junction proteins	Lung cancer	[139]
Endothelial-to-mesenchymal transition	Zn(PipNONO)Cl	Inhibition of EndMT	Lung cancer	[146]

## Data Availability

Not applicable.

## Abbreviations

AKT	protein kinase B
ALCAM	activated leukocyte cell adhesion molecule
BBB	blood–brain barrier
CD	cluster of differentiation
CNS	central nervous system
COX2	cyclooxygenase-2
EndMT	endothelial-to-mesenchymal transition
ERK	extracellular signal-regulated kinase
GBP1	guanylate-binding protein 1
GLUT1	glucose transporter 1
HER2	human epidermal growth factor receptor 2
ICAM-1	intercellular adhesion molecule-1
IGF2	insulin-like growth factor 2
IgSF	immunoglobulin superfamily
IL	interleukin
ILK	integrin-linked kinase
JAK	janus kinase
L1CAM	cell adhesion molecule L1
LFA-1	lymphocyte function-associated antigen 1
MAPK	mitogen-activated protein kinase
Mfsd2a	major facilitator superfamily domain 2a
MIF	macrophage migration inhibitory factor
miR	micro-RNA
MMP	metalloproteinase
MRTF	myocardin-related transcription factor
NSCLC	non-small cell lung cancer
PECAM-1	platelet endothelial cell adhesion molecule 1
PSGL-1	platelet selectin glycoprotein ligand- 1
ROCK	Rho-associated protein kinase
SDF-1	stromal cell-derived factor 1
sLex	sialyl-Lewis X
SP	substance P
ST6GALNAC5	α2, 6-sialyltransferase
TEM	trans-endothelial migration
TNFα	tumor necrosis factor α
uPAR	urokinase plasminogen activator receptor
VEGF	vascular endothelial growth factor
STAT3	signal transducer and activator of transcription 3
3′UTR	3′untranslated region
AKR1B10	aldo-keto reductase family 1 B10
Ang-2	angiopoietin-2
BCRP	breast cancer resistance protein
BE	brain endothelium
BEC	brain endothelial cells
BM	brain metastasis
BTB	blood–tumor barrier
CCL	CC chemokine ligand
Cx	connexins
CXCL	chemokine (C-X-C motif) ligand
CXCR	chemokine receptor
DHA	docosahexaenoic acid
ECM	extracellular matrix
EMT	epithelial-mesenchymal transition
ER	estrogen
ESG	endothelial surface glycocalyx
FUT	fucosyltransferases
GEF-H1	guanine nucleotide exchange factor-H1
GFAP	glial fibrillary acidic protein
HA	hylarunan
HB-EGFHB-EGF	heparin-binding epidermal growth factor-like growth factor
HPSE	heparanase
HRG	heregulin
HSPG	heparan sulfate proteoglycan
JAM	unction adhesion molecules
MTf	melanotransferrin
MUC1	mucin-1
NRP1	neuropilin-1
PAI-1	plasminogen activator inhibitor-1
PDPK1	3-phosphoinositide-dependent protein kinase-1 gene
PI3K	hosphatidylinositol 3-kinase
PLEKHA5	pleckstrin homology domain-containing family A member 5
PR	progesterone receptors
RAGE	receptor for advanced glycation end product
S100A4	EF-hand calcium-binding protein
SCLC	small cell lung cancer
Sdcs-1	syndecan-1
Sema4D	semaphorin 4D
SMA	α-smooth muscle actin
TGF-β	transforming growth factor
VCAM-1	vascular cell adhesion protein 1
VE-cadherin	vascular endothelial cadherin
VLA-4	very late antigen-4
YAP	yes-associated protein
ZO	zonula occluden
